# Use of genetically modified bacteria for drug delivery in humans: Revisiting the safety aspect

**DOI:** 10.1038/s41598-017-02591-6

**Published:** 2017-05-23

**Authors:** Udo Wegmann, Ana Lucia Carvalho, Martin Stocks, Simon R. Carding

**Affiliations:** 1 Gut Health and Food Safety Research Programme, The Quadram Institute, Norwich Research Park, Norwich, NR4 7UA Norfolk UK; 2 Norwich Medical School, University of East Anglia, Norwich Research Park, Norwich, NR4 7TJ Norfolk UK; 3 Plant Biotechnology Ltd., Norwich Research Park, Norwich, NR4 7UH Norfolk UK

## Abstract

The use of live, genetically modified bacteria as delivery vehicles for biologics is of considerable interest scientifically and has attracted significant commercial investment. We have pioneered the use of the commensal gut bacterium *Bacteroides ovatus* for the oral delivery of therapeutics to the gastrointestinal tract. Here we report on our investigations of the biological safety of engineered *B*. *ovatus* bacteria that includes the use of thymineless death as a containment strategy and the potential for the spread of transgenes *in vivo* in the mammalian gastrointestinal tract. We demonstrate the ability of GM-strains of *Bacteroides* to survive thymine starvation and overcome it through the exchange of genetic material. We also provide evidence for horizontal gene transfer in the mammalian gastrointestinal tract resulting in transgene-carrying wild type bacteria. These findings sound a strong note of caution on the employment of live genetically modified bacteria for the delivery of biologics.

## Introduction

The use of live genetically modified bacteria as delivery vehicles for the *in situ* delivery of therapeutics has to date relied on the use of specific commensal or food-grade bacteria, typically lactic acid bacteria, for heterologous gene expression. Efficacy has been demonstrated in proof of principle animal models of infectious and autoimmune diseases^1–3^ leading to the development of a number of sophisticated heterologous gene expression and genetic engineering systems^4–6^. This has culminated in clinical trials of GM-*Lactococcus lactis* ssp. *cremoris*, engineered to secrete the anti-inflammatory cytokine interleukin-10 in inflammatory bowel disease patients^7^. We have focused on the development of a novel protein delivery technology using the anaerobic Gram-negative human gut commensal bacterium *Bacteroides ovatus* for the diet-controlled delivery of therapeutic proteins to the gastrointestinal tract^8–10^. Following successful proof of principle studies using GM-*B*. *ovatus* engineered to secrete human cytokines for the treatment of experimental intestinal colitis^8^, the GM-strains were re-engineered to make them suitable for first-in-man studies. Apart from technical feasibility and clinical safety a major issue that all technologies of this kind must fully address is the biological safety of the genetically modified bacteria. Specifically, ensuring environmental containment and preventing the spread of the transgene by horizontal gene transfer as well as the survival of the genetically modified bacteria in the environment.

## Results and Discussion

### *In vitro* and *in vivo* horizontal gene transfer in *Bacteroides ovatus*

Thymineless death (TLD), the fatal process that thymine auxotrophic microorganisms undergo in response to thymine starvation, has previously been used as a biosafety strategy in for example, GM-*L*. *lactis* strains^11^. It was first discovered in *E*. *coli* ^12^ and exists in many other organisms including eukaryotes^13^. We reasoned that incorporating thymine auxotrophy, as employed for GM-*L*. *lactis* in a phase I clinical trial^7^, in addition to the anaerobic nature of *B*. *ovatus*, seemed an appropriate environmental containment strategy to adopt. A further advantage of using thymidine auxothrophy is that it facilitates a *thyA* based counter-selectable gene deletion and integration system for *B*. *ovatus*, similar to that described for *B*. *fragilis* ^14^.

**Figure 1:**
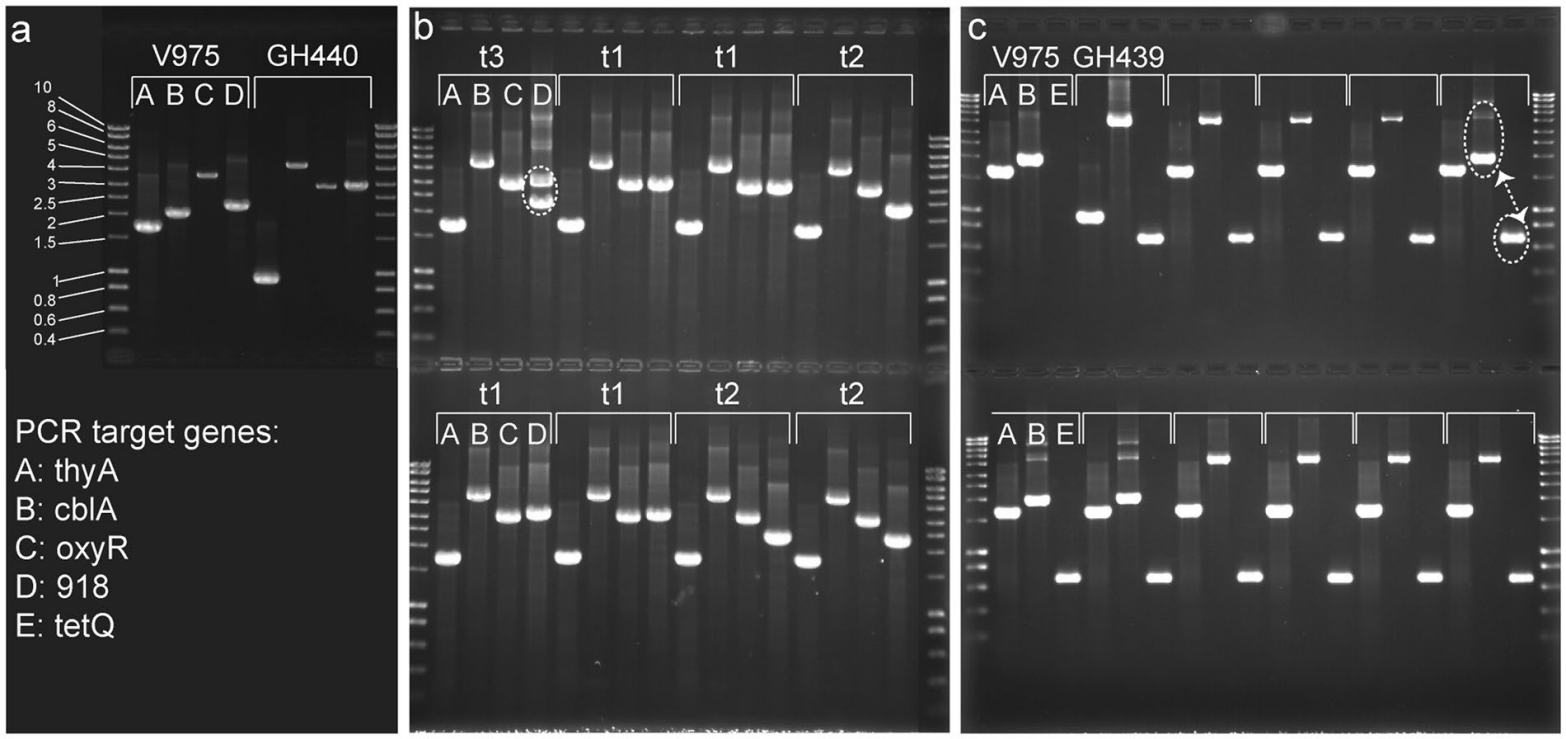
(**a**,**b**) Amplicon patterns of donor and recipient stains (**a**) and isolates after filter mating experiments (**b**). (**c**) Amplicon patterns of donor and recipient stains and isolates from the murine GI tract. A, B, C, D and E indicate the target (primer pairs) used in PCR, t denominates the different types (1–3) observed. The brackets above indicate tracks that belong to a specific isolate. Patterns indicating potential gene duplication events are circled. The arrow highlights the simultaneous presence of the wt *cblA* gene and the *tetR* gene in this isolate.

**Figure 2:**
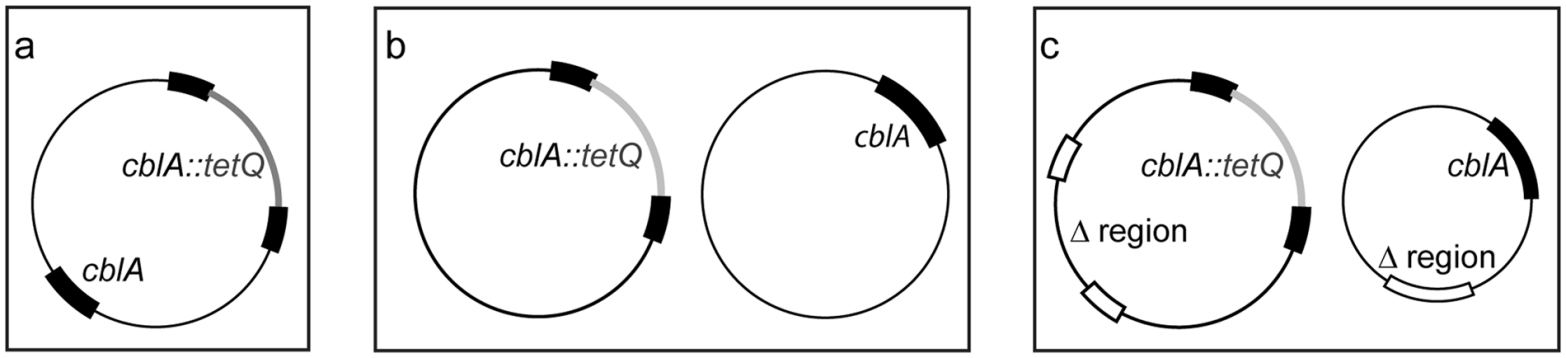
Schematic representation of 3 possible *cblA* gene duplication scenarios: (**a**) insertion of duplicated *cblA* gene into random position of chromosome, (**b**) diploid bacteria carrying 2 identical chromosomes differing only in their respective *cblA* allele, (**c**) partially diploid bacteria due to partial deletion from the 2^nd^ chromosome with the chromosomes differing in their respective *cblA* allele, the gene content and synteny.

Horizontal gene transfer in prokaryotes occurs via transformation, conjugation, or transduction^15^. As we were not aware of any reports of transduction or the occurrence of natural competence in *Bacteroides*, and since conjugation involving *E*. *coli* helper strains is routinely employed to genetically manipulate *Bacteroides* species we focused on conjugation as a potential mechanism of horizontal gene transfer. Two *B*. *ovatus thyA* deletion strains (see methods) tagged with the *tetQ* marker gene, GH439 (*ΔthyA*, *cblA::tetQ*) and GH440 (*ΔthyA*, *cblA::tetQ*, *ΔoxyR*, *BACOV975_00918::tgfβ_ex1*) were created (Table 1). To our knowledge a high-frequency of recombination (Hfr) phenotype involving the conjugational transfer of the whole chromosome from one cell to another has not been observed in *Bacteroides* species and as the only Hfr-type transfer reported to date relates to the partial transfer of the conjugative transposon CTnERL by *B*. *thetaiotaomicron* ^16^, we did not expect to detect chromosomal gene transfer events. In contrast, Hfr-type conjugation including the transfer of the *thyA* gene is well documented in *E*. *coli* ^17^ and, owing to the lactococcal sex factor, can occur in *L*. *lactis* ^18, 19^. Initially horizontal gene transfer was examined *in vitro* using *B*. *ovatus* V975 and GH439 or GH440 (Table 1) as potential *B*. *ovatus* donor and recipient cells during filter mating experiments in the absence of *E*. *coli* helper cells. Transconjugant colonies displaying a *thyA* ^+^/*tet* ^*R*^ phenotype were readily detected at a frequency of 0.9–4.2 × 10^−7^ transconjugants per recipient with the frequency independent of tetracycline use. PCR confirmed the genotype of donor and recipient cells as *thyA* ^+^/*cblA* (wt) and, *ΔthyA*/*cblA::tetQ* (GH439) or *ΔthyA*/*cblA::tetQ*/*ΔoxyR*/*BACOV975_00918::tgfβ* (GH440), respectively (Fig. 1a; GH439 and data not shown; Table 2). Horizontal gene transfer in the corresponding transconjugants was confirmed by PCR and their genotypes were determined as *thyA* ^+^/*cblA::tetQ* (conjugation with GH439, data not shown; Table 2) and *thyA* ^+^/*cblA::tetQ*/*ΔoxyR*/*BACOV975_00918::tgfβ* (type1), *thyA* ^+^/*cblA::tetQ*/*ΔoxyR*/*BACOV975_00918* (type 2) or *thyA* ^+^/*cblA::tetQ*/*ΔoxyR*/*BACOV975_00918*/*BACOV975_00918::tgfβ* (type 3) (conjugation with GH440, Fig. 1b, Table 2). The latter genotype, as indicated by fragments of 2250 bp and 3019 bp corresponding to the *BACOV975_00918 *wt gene as well as the *BACOV975_00918::tgfβ* locus (Fig. 1b), suggested a possible *BACOV975_00918* gene duplication event. Since strains GH439 and GH440 are derivatives of *B*. *ovatus* V975 and virtually genetically identical we were unable to ascertain which strains acted as donor. However, it is reasonable to assume that all three strains share this ability and as a result act simultaneously as recipient and donor. To determine whether horizontal gene transfer occurred *in vivo* in the mammalian gastrointestinal tract mice were orally gavaged with wt and GH439 bacteria sequentially with a one hour interval and feces subsequently sampled, homogenized and plated on selective media with potential transconjugants identified by PCR (see methods). Horizontal gene transfer events were identified in 5/10 colonized mice from which 48 *thyA* ^+^/*tet* ^*R*^ colonies were obtained from fresh feces with 21 assessed by PCR. In all cases horizontal gene transfer was confirmed on the basis of amplicon patterns (Fig. 1) indicative of a *thyA* ^+^/*tet* ^*R*^ genotype, and phenotypically by the growth of clones in liquid cultures in the presence of tetracycline without thymidine. As seen in the *in vitro* experiment for *BACOV975_00918*, some clones isolated from mouse feces produced a *cblA* amplicon pattern (see Fig. 1c, Table 2) indicative of a duplication of the β-lactamase gene with a 2040 bp amplicon representing the wt *cblA* allele as well as a 4206 bp amplicon expected for the *cbl::tetQ* allele and the *tetQ* specific 675 bp amplification product. These isolates could be grown in liquid culture without thymidine in the presence of ampicillin (200 μg/ml) and tetracycline (5 μg/ml). As these transconjugants had arisen from a horizontal gene transfer event that had taken place in the murine GI-tract, one of the isolates, number 4, was investigated further. Southern blot analysis of AvaI-restricted chromosomal transconjugant 4 DNA using a *cblA* and a *tetQ* specific probe revealed a banding pattern consistent with the presence of two *cblA* loci, one representing the wt and the other the *cblA::tetQ* topography (data not shown). Several scenarios could explain this result, the most likely of which is the random insertion of one of the alleles into the chromosome (Fig. 2a) creating a similar sized fragment in the Southern blot. Alternatively, two chromosomes may exist, each carrying one of the two alleles. In this scenario the chromosomes could either be identical bar their respective *cblA* loci (Fig. 2b) or, one could be smaller (Fig. 2c) due to a deletion, representing only a part of the chromosome. However, bacteria unlike higher eukaryotes, are generally haploid^20^ and contain a single chromosome, which is replicated during the cell cycle. Some exceptions have been reported^21, 22^ and mini-chromosomes have been genetically engineered for *E*. *coli* ^23^, although these rely on antibiotic resistance markers to be stably maintained. To distinguish these possibilities, the entire genome of transconjugant 4 was sequenced. We predicted that in the case where the *cblA* gene would have been randomly integrated into the chromosome two additional contigs would be found one representing the *cblA::tetQ* locus and the other the *cblA* gene in its new genetic location. In the case of an additional smaller chromosome, a contig joining genetic locations that are not adjacent on the wt chromosome would be present in addition to a contig representing the *cblA::tetQ* locus. In the case of two virtually identical chromosomes, we would expect all contigs to align to the wt sequence bar that representing the *cblA::tetQ* locus. After assembling 5.2 million paired end reads equivalent to >200-fold coverage of the wt genome into 100 contigs of ≥150 bp using the SPAdes^24^ software, contigs of ≥150 bp were aligned to the wt sequence^25^ either manually in a STADEN^26^ assembly or computationally using QUAST^27^; QUAST revealed that the contigs in the assembly represented 98.3% of the original sequence, with NG75 and LG75 values of 162375 bp and 16, respectively. Three small insertions of 63, 204 and 313 bp and two small local inversions of 1.2 and 7.2 kb, respectively, were identified in the transconjugant 4 assembly. Only two contigs could not be aligned to the wt genome. The first (151 bp) represented part of an insertion sequence (IS) element with its existence in *B*. *ovatus* V975, GH439 and transconjugant 4 confirmed by PCR, excluding it arising from the formation of a smaller chromosome. The second contained the *cblA::tetQ* locus, with sequences of this contig either side of the *tetQ* insertion being aligned perfectly to the wt sequence, confirming the Southern blot finding that transconjugant 4 harbored two chromosomes that were identical apart from their *cblA* locus and hence are at least diploid. We do not know whether *B*. *ovatus* is generally diploid, but it is clear it can use this ability to acquire new genes, which in turn aids in circumventing genetically engineered safety measures, for example, by maintaining a modified *thyA* locus whilst acquiring a functional wt gene. In this context it is noteworthy that several *L*. *lactis* subsp. *cremoris* and *lactis* strains, including MG1363, a derivative of which was used in a phase I clinical trial involving live bacteria, have been described as diploid^28^.

**Table 1:** Bacterial Strains.

Strain	Description	Reference
*Bacteroides ovatus* V975	Wild type	*B*. *ovatus* strain V975 was obtained from T.R. Whitehead (USDA ARS NCAUR, Peoria, IL)
*Bacteroides ovatus* GH439	*ΔthyA*, *cblA::tetQ*	This work
*Bacteroides ovatus* GH440	*ΔthyA*, *cblA::tetQ*, *ΔoxyR*, *BACOV975_00918::tgfβ_ex1*	This work
*E*. *coli* strain HB101 pRK2013^29^	F–, *thi*-1, *hsdS*20 (r_B_^−^, m_B_^−^), *supE*44, *rec*A13, *ara*14, *leuB*6, *proA*2, *lacY*1, *galK*2, *rpsL*20 (str^r^), *xyl*-5, *mtl*-1. pRK2013 (*KmR*, oriColE1, RK2-Mob+, RK2-Tra+)	Clontech
*E*. *coli* GC10	F^−^, *mcrA*, ∆(*mrr-hsdRMS*-*mcrBC*), Φ80d, *lacZ*∆M15, ∆lacX74, endA1, *recA*1, ∆(*ara*, *leu*) 7697*araD*139 *galU*, *galK*, *nupG*, *rpsL*, λ^−^, T1R.	GeneChoice, Inc.

**Table 2:** PCR amplicon sizes.

*B*. *ovatus* strains			
Primers	V975	GH439	GH440
	Expected fragment sizes		
ΔthyA_folA_R/thyA_F (A)	1699	909	909
cblA_F/cblA_R (B)	2040	4206	4206
oxyR left_flank/oxyR_r_flank3 (C)	3484	ND	2946
918_left_flank/918_right_flank (D)	2250	NA	3019
RT_tetQ_3′/RT-tetQ_5′ (E)	NA	673	673

### *In vitro* and *in vivo* viability of *Bacteroides ovatus thyA* mutants

**Figure 3:**
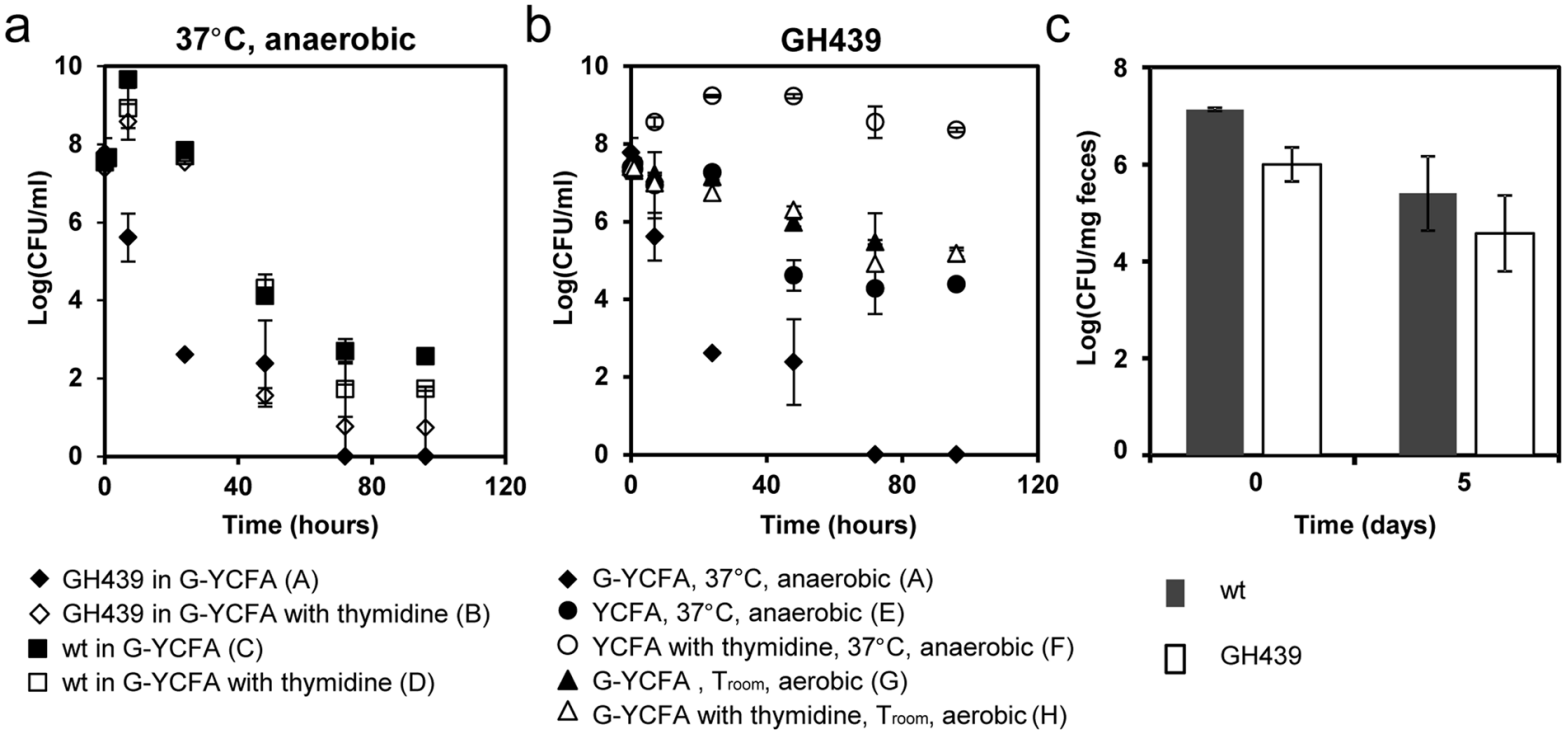
Growth and survival of *B*. *ovatus* wt and GH439 strains. (**a**) Growth and survival of wt and GH439 under optimal growing conditions with and without thymidine. (**b**) Growth and survival of GH439 under different environmental condition with and without thymidine. (**c**) Survival of wt and GH439 in mouse fecal samples prior to (0) and at 5 days post inoculation. In (**a**,**b**) data shown are representative of at least two independent experiments. In (**c**) values represent the mean of data from three independent experiments. Error bars indicate standard deviation.

As expected the viability of GH439 was similar to that of the wt when grown in the presence of thymidine (Fig. 3a, condition B and D). The same growth profile was observed for the wt strain in the absence of thymidine (Fig. 3a, condition C) whereas the viability of GH439 in the absence of thymidine declined immediately after inoculation (Fig. 3a, condition A) with 1% viability after 7 h and 0.001% after 24 h. This contrasted with the wt strain which, under the same conditions, showed no decline in viability over 24 h (Fig. 3a which shows that viability of cells in condition A is significantly lower than that of conditions B, C and D, at 7 and 24 h; 2-way ANOVA, p < 0.01). The rapid decline in viability seen after inoculation of the *B*. *ovatus thyA* deletion strain was not observed when bacteria were cultured under non-growing conditions and when thymidine was absent (Fig. 3b, which shows that viability of cells in condition A is significantly lower than that of conditions E, F, G, H, after 24 h; 2-way ANOVA, p < 0.01). Indeed, when GH439 bacteria lacked glucose as a carbon source (Fig. 3b, conditions E and F) the cells remained almost 100% viable after 24 h and by 72 h had retained more than 1% viability, in contrast to GH439 cells under growing conditions but lacking thymidine, which were no longer viable after 72 h (Fig. 3b, conditions A). The viability of GH439 cells inoculated under optimal environmental conditions (with thymidine but without glucose) increased slightly during the first 24 h, unlike the cells in the same conditions but without thymidine (Fig. 3b, conditions E and F). This is because YCFA is not a minimal medium and *B*. *ovatus* does not grow in minimal medium, with the yeast extract present in the medium (0.05%) most likely supporting some limited growth when thymidine is available. When GH439 cells were inoculated in medium with glucose but under non-optimal temperature and oxygen conditions, the profile was similar, independent of the presence of thymidine. Approximately 100% of cells retained their viability during the first 24 h, which then decreased slowly, probably due to oxygen stress, and after 96 h (4 days) approximately 1% of cells retained viability (Fig. 3b, conditions G and H). Thus, culture conditions that prevent bacterial growth protect bacteria from TLD.

To determine whether *B*. *ovatus* TLD occurs *in vivo*, mice were colonized with wt or GH439 bacteria their feces collected and bacterial cfu determined. The number of viable wt and GH439 bacteria after 5 days of storage of the fecal samples at ambient temperature and in the presence of oxygen were 3.9% and 6.6% of the initial counts, respectively (Fig. 3c), indicating that *B*. *ovatus thyA* deletion strains survive and can be readily acquired by a new host. Furthermore, TLD is entirely overcome through the exchange of genetic material between wt and GM bacteria, an occurrence that could also result in the escape of the transgene into an unintended population of bacteria.

Based on these results we conclude that TLD is unsuitable as a biosafety control measure in *Bacteroides* species and we therefore caution against its use in other bacterial species. Indeed, our work brings into sharp focus the uncertainty all biosafety control measures suffer from, in that whilst it is possible to define the genetic status of the genetically modified bacteria prior to delivery into a host, it is not possible to completely predict or control what type of bacteria are already present or enter the system at a later stage nor how the GM bacteria interact with other bacteria in their environment and how this impacts on their genetic stability and ability to escape any containment measures.

## Material and Methods

### Bacterial growth conditions and transformations

*Escherichia coli* strains were grown in Luria-Bertani medium at 37 °C. *B*. *ovatus* V975 and derivative strains (Table 1) were grown under anaerobic conditions at 37 °C in BHI medium (Oxoid, UK) supplemented with 0.001% haemin (BHIH) and 50 μg/ml thymidine when necessary. Antibiotics were added as selective agents when appropriate; erythromycin 5 μg/ml, kanamycin 50 μg/ml, rifampicin 35 μg/ml, spectinomycin 100 μg/ml, tetracycline 5 μg/ml and trimethoprim 100 μg/ml. *E*. *coli* was transformed by electroporation using a Gene Pulser II (Bio-Rad, UK). The construction of plasmids described below was performed using *E*. *coli* GC10 as the host strain. Plasmids were mobilized into *B*. *ovatus* V975 using the *E*. *coli* strain HB101 pRK2013^29^ as a helper strain for triparental filter matings^30^.

### Construction of *B*. *ovatus* mutants

To establish a counter-selectable gene deletion and integration system in *B*. *ovatus* first, the *thyA* gene was deleted from the chromosome of this strain, to enable counter-selection with trimethoprim. For this purpose a DNA fragment representing the *thyA* containing region of the *B*. *ovatus* chromosome void of *thyA* was created through splice overlap extension PCR. Firstly, the primer pairs ΔthyA_cls_F/ΔthyA_cls_R and ΔthyA_folA_F/ΔthyA_folA_R (see supplementary Table 1 for all PCR primers in study)were used with *B*. *ovatus* V975 genomic DNA as template to generate the respective PCR fragments. Second, the PCR products were used as templates for the splice PCR involving the primer pair ΔthyA_cls_F/ΔthyA_folA_R. The resulting 1595 bp product was cloned into the SmaI site of the suicide vector pFD516^31^ creating plasmid pGH138, which was conjugated into *B*. *ovatus*. Single-crossover transconjugants were selected on erythromycin (1 μg/ml) and subsequently plated onto BHIH agar containing thymidine and trimethoprim to select for the *thyA* gene deletion. The resulting colonies were screened by PCR using primer pair ΔthyA_left_flank/ΔthyA_right_flank and the resulting PCR product from a positive clone was sequenced in its entirety to confirm the integrity of the resulting *thyA* deletion strain denominated GH369. To allow subsequent cycles of gene deletions or insertions the suicide vector pGH139 was constructed by inserting a 905 bp PCR fragment carrying the *thyA* gene amplified with primer pair thyA_F/thyA_R into PshAI/ScaI restricted pFD516. Plasmid pGH139 allows for the selection of single-crossover transconjugants on BHIH agar upon conjugation into *B*. *ovatus* GH369 and the subsequent counter-selection on BHIH plates containing thymidine and trimethoprim to identify clones in which a double-crossover event has taken place. Using this system the tetracycline resistant *B*. *ovatus* GH439 was created as follows. The *cblA* integration target site was amplified using primer pair cblA_F/cblA_R and the resulting 2040 bp product was cloned into PvuII restricted pGH139. Next, the resulting plasmid was restricted with EcoRI and a *tetQ* carrying fragment, generated through PCR using primer pair tetQ_MfeI_F/tetQ_MfeI_R and pFI2716^32^ as template, was restricted with MfeI and ligated into the EcoRI site generating pGH139_cblA_tetQ. This plasmid was then conjugated into *B*. *ovatus* GH369 and *tetQ*-tagged double-crossover transconjugants were isolated in a two-step process as described above. A second integration plasmid was created in a similar fashion using the primer pair 918_F/918_R and the resulting 2075 bp product was cloned into the SmaI site of pGH139 and the intrinsic EcoRV site was used as the cloning site for the *tgfβ_ex1* gene acquired through gene synthesis, coding for the active form of the human transforming growth factor beta-1 (position 279–390) fused to the *Bacteroides fragilis* enterotoxin signal peptide. The resulting plasmid was employed to generate *B*. *ovatus* GH378 from GH369. To delete the *oxyR* gene from the chromosome of *B*. *ovatus* a 2661 bp PCR fragment generated with primer pair ΔoxyR_F/ΔoxyR_R was cloned into PvuII restricted pGH139 and subsequently the internal PvuII/NruI fragment was deleted from the *oxyR* gene resulting in pGH148, which in turn was used to generate GH397 from GH378. Finally pGH139_cblA_tetQ was used to generate *B*. *ovatus* GH440 from GH397.

### Filter mating experiments

Overnight cultures of strains were grown anaerobically in BHIH containing thymidine and in the case of *B*. *ovatus* GH439 and GH440 with and without tetracycline. Cultures were then inoculated 1/20 into 20 ml BHIH containing thymidine and incubated anaerobically for 3.5 h. Subsequently donor and recipient cultures were united in 50 ml tubes, mixed and spun down at ambient temperature and 5000 g for 30 min. Culture supernatants were removed, cells were resuspended in the same media, transferred onto a 0.45 μm filter disc located on a BHIS agar plate containing thymidine and incubated anaerobically overnight. To enumerate the conjugation efficiency, cells were washed off the filter and serial dilutions plated or spotted onto either BHIS agar (V975), BHIS agar containing thymidine and tetracycline (GH439, GH440) or, BHIS agar containing tetracycline and rifampicin (transconjugants).

### Animal studies

Seven-week-old male C57BL/6J mice were housed in a conventional animal facility and fed standard laboratory chow. Prior to administering *B*. *ovatus* strains, mice (two groups of 3) were treated for 7 days with 1 mg/ml ampicillin and 1 mg/ml neomycin via drinking water. Bacteria suspensions of *B*. *ovatus* GH439 (Δ*thyA*, *cblA::tetQ*) and *B*. *ovatus* V975 (wt) containing 5 × 10^8^ CFU were administrated sequentially by orogastric gavage with a 1 h interval between gavages. Animal experiments were conducted in full accordance with the Animal Scientific Procedures Act 1986 under Home Office approval. Fresh feces were collected into sterile containers, weighed and homogenized in PBS. Serial dilutions of the suspensions were plated onto BHIH-RNAG (BHIH plates supplemented with 35 µg/ml rifampicin, 10 µg/ml neomycin, 75 µg/ml amikacin and 100 µg/ml gentamycin) to select for the *B*. *ovatus* V975, BHIH-ThymidTRNAG (plates supplemented with 50 µg/ml thymidine, 5 µg/ml tetracycline, 35 µg/ml rifampicin, 10 µg/ml neomycin, 75 µg/ml amikacin and 100 µg/ml gentamycin) was used to select for *B*. *ovatus* GH439, and BHIH-TRNAG (plates supplemented with 1.5 µg/ml tetracycline, 35 µg/ml rifampicin, 10 µg/ml neomycin, 75 µg/ml amikacin and 100 µg/ml gentamycin) to select for cells in cases of horizontal gene transfer. The plates were incubated anaerobically at 37 °C and CFU were counted 48 h later.

### PCR screening for horizontal gene transfer of *thyA*, *tetQ*, *BACOV975_00918* and *oxyR* after filter mating

Colonies obtained on BHIS agar containing tetracycline and rifampicin were assessed by colony PCR by suspending single colonies in 30 µl of sterile water and using 1 µl of this cell suspension in a 25 µl PCR reaction using GoTaq Green Master Mix (Promega) and primer pairs ΔthyA_folA_R/ΔthyA_F (a), cblA_F/cblA_R (b), oxyR left_flank/oxyR_r_flank3 (c) or 918_left_flank/918_right_flank (d) according to the manufacturer’s instruction. The resulting amplicons (Table 2) were analyzed and compared by gel electrophoresis to amplicon patterns obtained with DNA isolated from donor and recipient cultures grown for the filter mating experiment.

### Screening for horizontal gene transfer of *tetQ and thyA* in the murine colon by PCR

Colonies obtained on BHIH-TRNAG plates were streaked onto BHIH-TRNAG plates containing 5 µg/ml tetracycline and incubated anaerobically at 37 °C. Single colonies were either analyzed by colony PCR as described above or transferred into BHIH medium supplemented with tetracycline and total DNA was extracted from the cultures 24 h later using the GeneJET Genomic DNA Purification Kit (ThermoFisher) according to the manufacturer’s instructions. To determine the genotype of the bacterial isolates with regards to *thyA*, *cblA and tetQ*, PCRs using cell suspensions or genomic DNA as template were carried out using primer pairs ΔthyA_folA_R/thyA_F (a), cblA_F/cblA_R (b) and RT_tetQ_3′/RT-tetQ_5′ (e) and the resulting amplicons (Table 2) analyzed by gel electrophoresis and compared to amplicon patterns obtained with DNA isolated from *B*. *ovatus* V975 and GH439 cultures grown in parallel.

### Viability tests

*B*. *ovatus* wt and GH439 strains were grown over-night at 37 °C under anaerobic conditions, in YCFA medium supplemented with 0.5% glucose (G-YCFA) and with 50 µg/ml thymidine in the case of GH439, washed once with PBS and diluted (1/100) in YCFA or G-YCFA. YCFA and G-YCFA cultures of wt and GH439 were kept at 37 °C under anaerobic conditions or at ambient temperature under aerobic conditions. When indicated, wt and GH439 cultures were supplemented with 50 µg/ml thymidine. At different time points samples were taken diluted in PBS and plated in BHI (wt) or BHI supplemented with 50 µg/ml thymidine (GH439). The plates were incubated anaerobically at 37 °C and CFU counted 48 h later.

## Electronic supplementary material

Dataset 1
