# Supplementary material for: Use of genetically modified bacteria for drug delivery in humans: Revisiting the safety aspect
Source: Sci Rep. 2017 May 23;7:2294. doi: 10.1038/s41598-017-02591-6 (PMC5442108; doi:10.1038/s41598-017-02591-6)
Supplement: Supplementary file 1 — Dataset 1 [file 41598_2017_2591_MOESM1_ESM.doc]

**Use of genetically modified bacteria for drug delivery in humans: Revisiting the safety aspect**

Udo Wegman, Ana Lucia Carvalho, Martin Stocks and Simon R Carding

**Supplementary Table 1. Primers used in the study**

| **Primer** | **Sequence** |
| --- | --- |
| ΔthyA_cls_F | AGGCGGAATGAATATCGCGG |
| ΔthyA_cls_R | CTCATCTTTTTTAATAATTAATAAGTTGCATAATCTGTG |
| ΔthyA_folA_F | AATTATTAAAAAAGATGAGTAAAGTATCAATC |
| ΔthyA_folA_R | ATCGGATATGAGACTTGCGC |
| ΔthyA_left_flank | CCCGTTCACCTTCCCTTATATC |
| ΔthyA_right_flank | GGCGCTGCGATCCATCAG |
| thyA_F | AGCGTTCTCCATGGAAGAAGTTCG |
| thyA_R | TTACACGGCTACTGCTCCTGC |
| tetQ_MfeI_F | TACTTACCAATTGAACCTACG |
| tetQ_MfeI_R | ATAGCAATTGCCTGACCGCTCC |
| ΔoxyR_F | GATGGTGAGGAAGTATCACAAC |
| ΔoxyR_R | ACAATGCTTCTCCTCCTTGG |
| cblA_F | GGATAACCATCTGACAATTCC |
| cblA_R | AAGCGAAGAACGTTTCTGC |
| oxyR left_flank | ATTCGTGATACTACAGTGAATG |
| oxyR_r_flank3 | TGAACATGCGAAACACACTC |
| 918_left_flank | CATCTGACCGATAGATTTGC |
| 918_right_flank | TGTTCATCAAACGCCTTCTC |
| RT-tetQ_3' | CAACAACTCATTGATACCGATA |
| RT-tetQ_5' | TGCTCACATTGATGCAGG |
